# Implications of inflammatory cell death-related IFNG and co-expressed RNAs (AC006369.1 and CCR7) in breast carcinoma prognosis, and anti-tumor immunity

**DOI:** 10.3389/fgene.2023.1112251

**Published:** 2023-06-20

**Authors:** Yongran Deng, Zhenlong Li, Mingmei Pan, Huayun Wu, Bingqiang Ni, Xueqiong Han

**Affiliations:** Department of Oncology, The Fifth Affiliated Hospital of Guangxi Medical University, Nanning, Guangxi, China

**Keywords:** breast carcinoma, IFNg, inflammatory cell death, AC006369.1, CCR7, prognosis, tumor microenvironment, immune checkpoint

## Abstract

**Objective:** Interferon-γ (IFN-γ) encoded by IFNG gene is a pleiotropic molecule linked with inflammatory cell death mechanisms. This work aimed to determine and characterize IFNG and co-expressed genes, and to define their implications in breast carcinoma (BRCA).

**Methods:** Transcriptome profiles of BRCA were retrospectively acquired from public datasets. Combination of differential expression analysis with WGCNA was conducted for selecting IFNG-co-expressed genes. A prognostic signature was generated through Cox regression approaches. The tumor microenvironment populations were inferred utilizing CIBERSORT. Epigenetic and epitranscriptomic mechanisms were also probed.

**Results:** IFNG was overexpressed in BRCA, and connected with prolonged overall survival and recurrence-free survival. Two IFNG-co-expressed RNAs (AC006369.1, and CCR7) constituted a prognostic model that acted as an independent risk factor. The nomogram composed of the model, TNM, stage, and new event owned the satisfying efficacy in BRCA prognostication. IFNG, AC006369.1, and CCR7 were closely linked with the tumor microenvironment components (e.g., macrophages, CD4/CD8 T cells, NK cells), and immune checkpoints (notably PD1/PD-L1). Somatic mutation frequencies were 6%, and 3% for CCR7, and IFNG, and high amplification potentially resulted in their overexpression in BRCA. Hypomethylated cg05224770 and cg07388018 were connected with IFNG and CCR7 upregulation, respectively. Additionally, transcription factors, RNA-binding proteins, and non-coding RNAs possibly regulated IFNG and co-expressed genes at the transcriptional and post-transcriptional levels.

**Conclusion:** Collectively, our work identifies IFNG and co-expressed genes as prognostic markers for BRCA, and as possible therapeutic targets for improving the efficacy of immunotherapy.

## Introduction

Breast carcinoma (BRCA) has a high incidence globally, with over two million cases per year (Sung et al., 2021). This malignancy represents a remarkable threat to female health and affects one in seven women over the course of a lifetime (Corti et al., 2022). Based upon the expression status of estrogen receptor (ER), progesterone receptor (PR), and human epidermal growth factor receptor 2 (HER2), four molecular subtypes have been widely accepted: luminal A, luminal B, HER2-enriched and basal-like tumors (Bidard et al., 2022; Curigliano et al., 2022; Shepherd et al., 2022). Despite the progress in early diagnosis and treatment, most patients still succumb to various complex malignant phenotypes (Martin et al., 2022; Mayer et al., 2022; Shepherd et al., 2022). Within 10 years following breast conservation surgical resection with post-operative radiotherapy, the recurrence rate is still as high as 3%–15% (Gadaleta et al., 2022). Emerging immunotherapy has exhibited promising results in BRCA, but with low response rates (Loibl et al., 2019; Schmid et al., 2020; Huober et al., 2022). Such alarming situation has prompted to determine innovative and effective therapeutic targets for BRCA.

Interferon-γ (IFN-γ) encoded by IFNG gene is the only member of the type II interferon family, which is an essential cytokine generated from activated T cells, natural killer (NK), and NK T cells in the tumor microenvironment (TME) (Dörrie et al., 1999; Wu H. et al., 2022; Wei et al., 2022). Cell death can provide host defense and maintain homeostasis (Niu et al., 2021; Wang Z. et al., 2022). IFN-γ can prime diverse inflammatory cell death mechanisms. For instance, IFN-γ secreted from CD8^+^ T cells rewires lipid metabolism of malignant cells through ACSL4, thus activating polyunsaturated fatty acids and sensitizing malignant cells to ferroptotic cell death (Liao et al., 2022). IFN-γ can also initiate macrophages for pathogen ligand-induced killing through caspase-8 and mitochondrial cell death signaling (Simpson et al., 2022). Moreover, the diverse implications of IFN-γ in BRCA (e.g., prognostication, therapeutic efficacy) have been demonstrated in prior studies (Witek Janusek et al., 2019). Non-etheless, IFN-γ-co-expressed genes and underlying molecular mechanisms remain indistinct in BRCA. For solving these problems, this work was implemented for determining and characterizing IFNG and co-expressed genes, and clarifying their implications in BRCA and probing possible epigenetic and epitranscriptomic mechanisms.

## Materials and methods

### Collection of BRCA datasets

BRCA transcriptome RNA-sequencing data (Htseq-FPKM) and matched clinical parameters were gathered from The Cancer Genome Atlas (TCGA) database. Somatic mutation, copy-number alteration, DNA methylation, and microRNA (miRNA) data were also extracted. External microarray datasets from the Gene Expression Omnibus database were online analyzed on the Kaplan-Meier Plotter platform.

### Selection of IFNG-co-expressed genes

Utilizing limma method (Ritchie et al., 2015), aberrant expressed genes in BRCA *versus* control specimens were selected with adjusted *p* < 0.05. Based upon the same threshold, genes with different expression between lowly and highly expressed IFNG BRCA were acquired. Above genes were intersected and named as BRCA- and IFNG-relevant genes. Next, weighted correlation network analysis (WGCNA) was implemented through WGCNA package (Langfelder and Horvath, 2008). Firstly, a clustering dendrogram was plotted, with removal of outliers via hierarchical clustering analysis. By Pearson’s test, interactions between genes were analyzed, and interaction pairs with *p* < 0.05 were used for constructing a similarity matrix. Afterwards, soft thresholding value was adopted for transforming the similarity matrix to the adjacency matrix. A scale-free network and topological overlap matrix were built, respectively. Next, a hierarchical clustering dendrogram was produced for detecting modules. At last, modules were merged with dynamic tree cutting approach. The module with the strongest connection to IFNG was chosen or subsequent analysis.

### Functional enrichment analysis

Enrichment on Gene Ontology (GO) or Kyoto Encyclopedia of Genes and Genomes (KEGG) pathways was analyzed based upon module genes by use of clusterProfiler approach (Yu et al., 2012).

### Cox regression analysis and nomogram establishment

Univariate-cox regression analysis on genes in the black module with prognosis was conducted. Genes with *p* < 0.05 were selected for the construction of a multivariate cox regression model. Based upon 1:1, TCGA-BRCA cases were randomized into the discovery and verification sets. Survival difference was then estimated. The predictive independency was analyzed utilizing cox regression analysis. A nomogram was defined with rms package, and predictive efficiency was demonstrated by calibration curves.

### Quantification of the TME components

CIBERSORT is an algorithm for characterization of the cellular compositions within bulk tissues based upon transcriptome profiling (Newman et al., 2015). The components within the TME were quantified by use of this algorithm.

### Genetic alteration assessment

Somatic variants were estimated by use of maftools package (Mayakonda et al., 2018). The mutated frequency of IFNG and co-expressed genes was extracted. GISTIC2.0 was adopted for copy-number alterations of above genes (Mermel et al., 2011).

### DNA methylation analysis

DNA methylation levels (beta-values) were normalized by use of preprocessCore package. Interactions of IFNG and co-expressed genes with methylation sites were then assessed.

### Non-coding RNA analysis

MiRNAs with different expression were screened between BRCA *versus* controls and lowly *versus* highly expressed IFNG BRCA following adjusted *p* < 0.05. Above miRNAs were intersected, and determined as BRCA- and IFNG-relevant miRNAs. Correlation analysis on long non-coding RNAs (lncRNAs) with IFNG and co-expressed genes was then carried out.

### Statistical analysis

For continuous variables, Student’s t-test, or one-way ANOVA test was utilized for comprising between groups. Chi-square or Fisher’s exact test was employed for analysis of categorical data. Kaplan-Meier curves of overall survival (OS) and recurrence-free survival (RFS) were plotted, with log-rank test for estimating survival difference. Correlation analysis was conducted with Pearson’s test. All analyses were achieved based upon the R platform (version 4.0.3). *p* < 0.05 indicated statistically significant.

## Results

### Expression and prognostic implication of IFNG and selection of IFNG-relevant genes in BRCA

**FIGURE 1:**
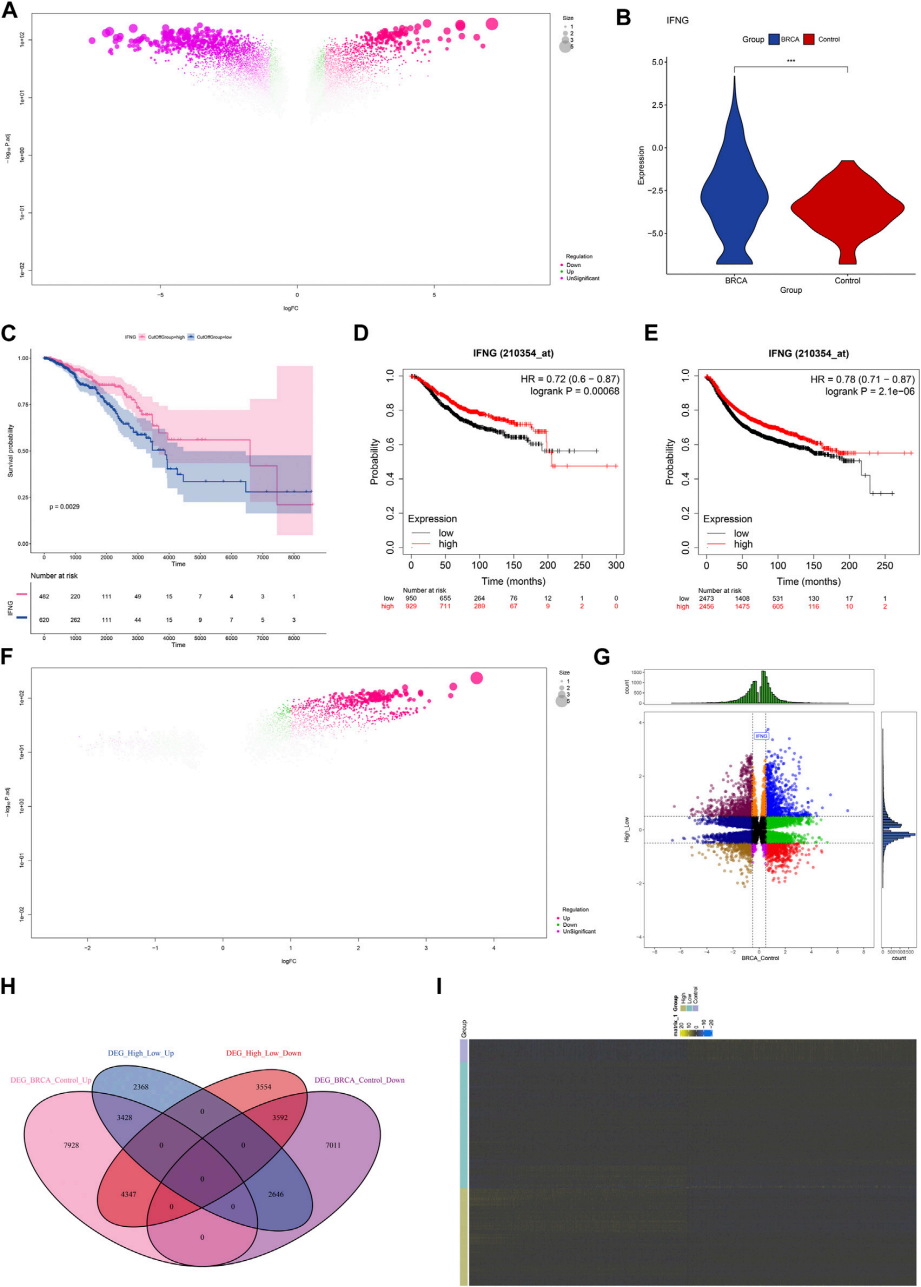
Expression and prognostic implication of IFNG and selection of IFNG-relevant genes in BRCA. **(A)** Genes with aberrant expression in BRCA *versus* control specimens. **(B)** IFNG transcript level in BRCA and controls. **(C)** OS probability of groups with low or high IFNG expression. **(D, E)** Verification of OS and RFS of two groups in multiple microarray datasets. **(F)** Genes with different expression in BRCA samples with low *versus* high IFNG expression. **(G, H)** The shared expression patterns in BRCA *versus* controls and high *versus* low IFNG expression. **(I)** The transcript level of IFNG-relevant genes in controls, BRCA with low or high IFNG expression.

The investigation on the transcriptional alterations in BRCA was conducted. With adjusted *p* < 0.05, 28,953 genes presented the differential expression in BRCA relative to controls (Figure 1A; Supplementary Table S1). Among them, we focused on IFNG that was prominently upregulated in BRCA (Figure 1B). Its prognostic significance was then evaluated. With the cutoff value, the classification of BRCA patients as low or high IFNG expression group was performed. As illustrated in Figure 1C, patients with high IFNG expression owned the notable survival superiority. The prognostic significance was further verified in multiple microarray datasets via the Kaplan-Meier Plotter. Consistently, IFNG upregulation was connected with better OS and RFS (Figures 1D, E). Above data unveiled the involvement of IFNG in BRCA pathogenesis. Afterwards, the relevant molecules of IFNG were probed. Consequently, 19,935 genes presenting different expression between low and high IFNG expression groups were selected (Figure 1F; Supplementary Table S2). After intersecting, 7020 IFNG-relevant genes were obtained (Figures 1G–I).

### Establishment of IFNG-based co-expression modules

**FIGURE 2:**
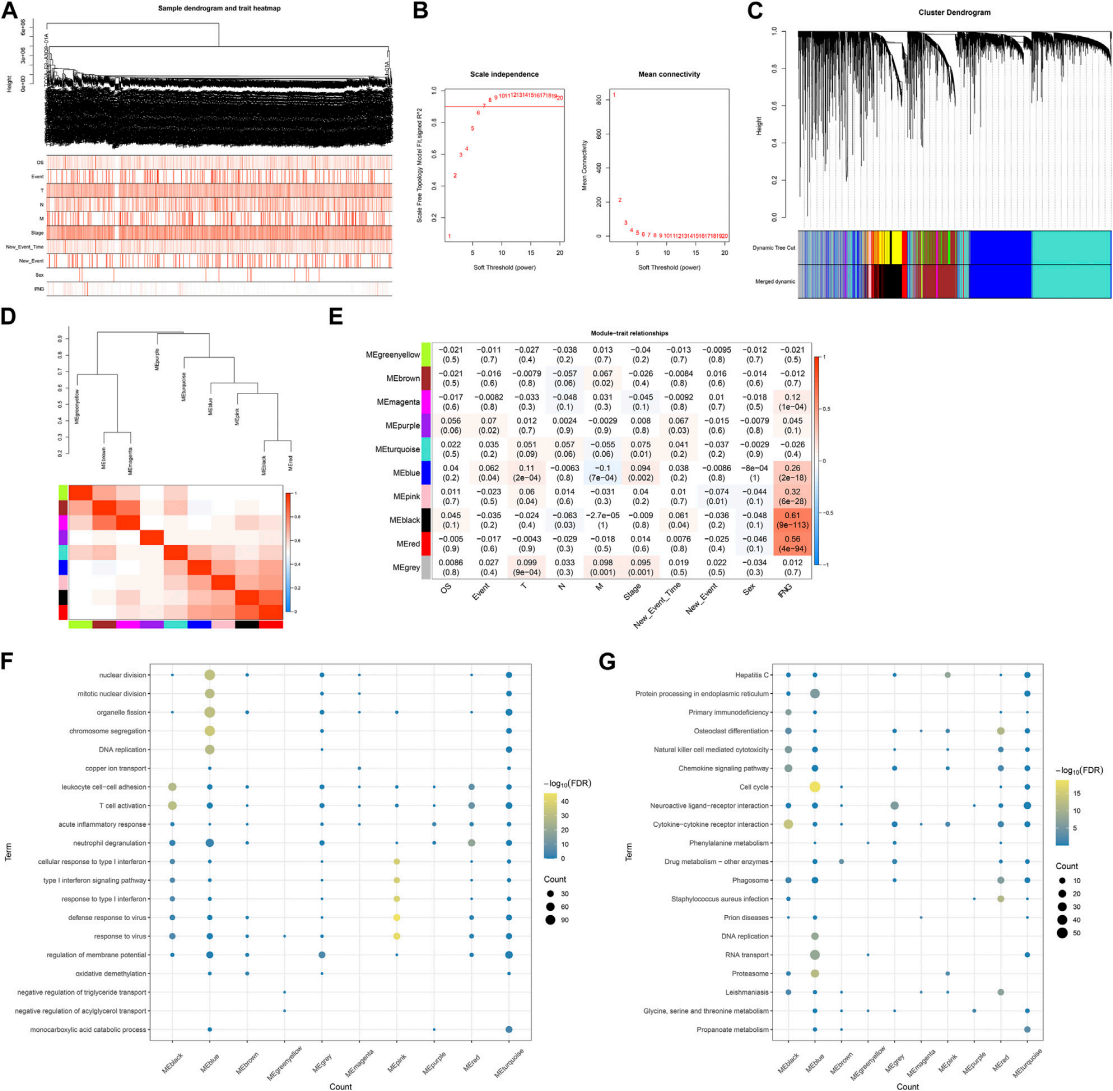
Establishment of IFNG-based co-expression modules. **(A)** Sample dendrogram and heatmap of clinical characteristics. **(B)** Scale independence along with mean connectivity under diverse soft-thresholding values. **(C)** Clustering dendrogram and merged modules. **(D)** Eigengene dendrogram and heatmap of eigengene adjacency. **(E)** Relationships of co-expression modules with clinical characteristics. **(F, G)** GO and KEGG pathways of genes in each module.

BRCA specimens with matched clinical and IFNG characteristics were included for WGCNA (Figure 2A). The appropriate soft-thresholding value was set as 6 through considering scale independence and mean connectivity (Figure 2B). Utilizing dynamic tree cutting method, highly connected genes were merged into ten modules (Figures 2C, D). Black module exhibited the strongest connection with IFNG (Figure 2E), which was regarded as IFNG-relevant module. It was noted that genes in the black module were prominently linked with immunity (e.g., T cell activation, leukocyte cell-cell adhesion, and cytokine-cytokine receptor interaction) (Figures 2F, G).

### Generation of an IFNG-co-expressed prognostic signature

**FIGURE 3:**
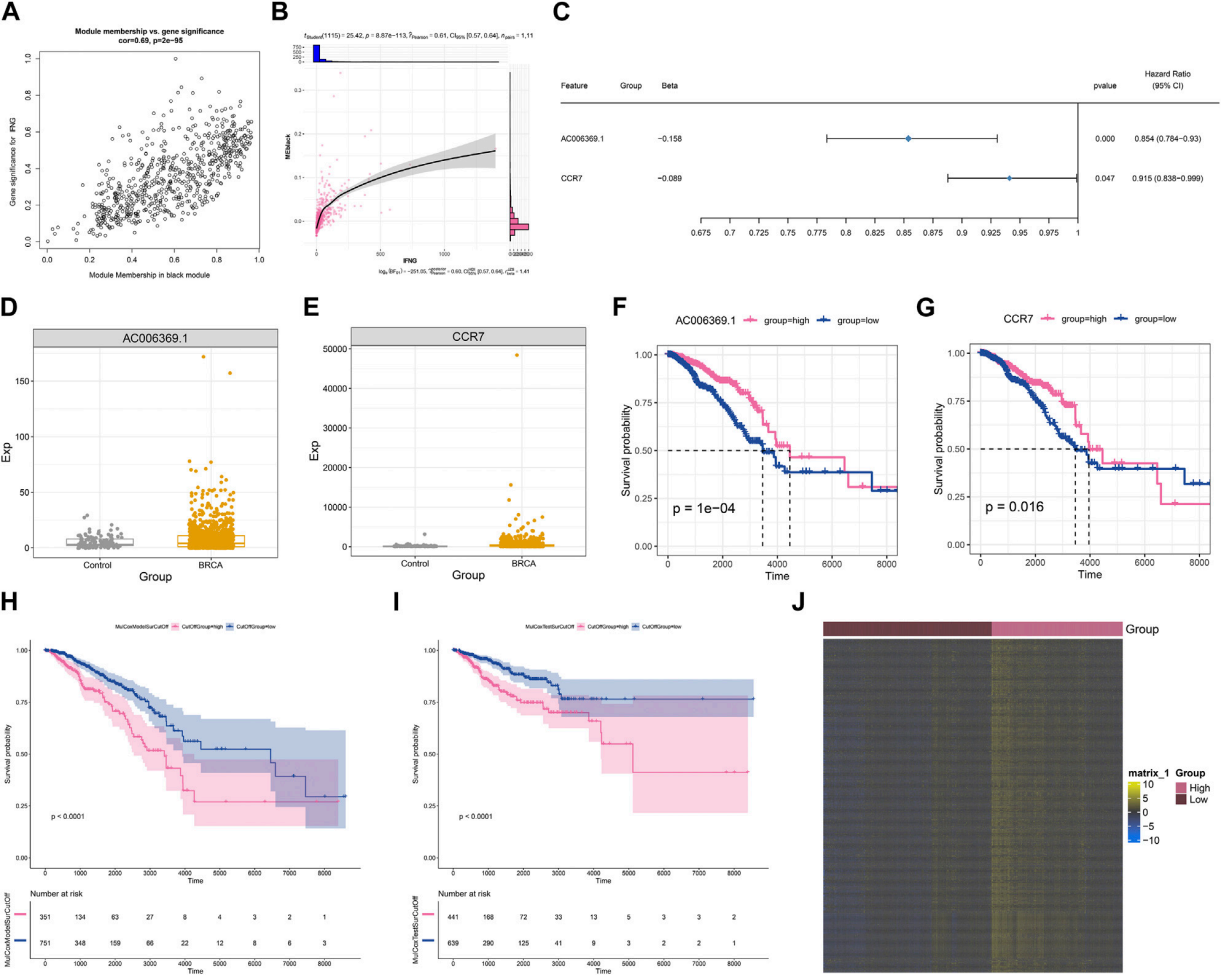
Generation of an IFNG-co-expressed prognostic signature. **(A)** Relationships of module membership in black module with gene significance for IFNG. **(B)** Correlation analysis on IFNG expression with black module. **(C)** Univariate-cox regression results of AC006369.1, and CCR7 with BRCA survival. **(D, E)** The transcript level of AC006369.1, and CCR7 in BRCA and controls. **(F, G)** OS analysis of AC006369.1, and CCR7 across BRCA patients. **(H, I)** OS difference between low- and high-score groups in the discovery and verification sets. **(J)** Heatmap of the expression patterns of IFNG-co-expressed genes in low- and high-score groups.

Module membership in black module exhibited a notably positive connection with gene significance for IFNG (Figure 3A). It was also demonstrated that black module was positively linked with IFNG (Figure 3B). Such evidence proved that genes in black module were IFNG-co-expressed genes. Most of them owned the significant survival significance of BRCA (Table 1). Notably, AC006369.1, and CCR7 presented the aberrant expression in BRCA *versus* controls, and their upregulation was in relation to OS outcomes (Figures 3C–G). They were incorporated into the multivariate-cox regression model, and worse OS was investigated in high-score patients both in the discovery and verification sets (Figures 3H, I). Most IFNG-co-expressed genes had the higher expression in high-than low-score groups (Figure 3J), indicating their subtype specific expression.

**TABLE 1:** Univariate-cox regression results of IFNG-relevant genes with BRCA prognosis.

IFNG-relevant genes	Beta	z	p	Hazard ratio	Lower	Upper
AC006369.1	−0.027	−2.67695	0.00743	0.973336	0.954265	0.992788
CCR7	0	2.671615	0.007549	1.000088	1.000023	1.000153
RPL4P1	−0.03	−2.44974	0.014296	0.970893	0.948216	0.994112
TRBV5.5	−0.045	−2.43696	0.014811	0.955617	0.921356	0.991154
TRDV1	−0.014	−2.42653	0.015244	0.985938	0.974724	0.997281
PSMB8	0	−2.36695	0.017935	0.999942	0.999894	0.99999
DEF6	0	−2.35957	0.018296	0.999729	0.999504	0.999954
SHISAL2A	−0.005	−2.23588	0.025359	0.9949	0.99045	0.999369
TRBC2	0	−2.19914	0.027868	0.999841	0.999699	0.999983
HCST	−0.001	−2.19251	0.028343	0.998989	0.998085	0.999893
GZMM	−0.002	−2.16979	0.030023	0.998335	0.996834	0.999839
RAC2	0	−2.08823	0.036778	0.999904	0.999814	0.999994
ARMH1	−0.002	−2.07626	0.037869	0.997758	0.995646	0.999874
IL12B	−0.006	−1.96702	0.049181	0.993893	0.987845	0.999978
TRBV4.2	−0.006	−1.92912	0.053717	0.993905	0.987751	1.000098
SPIB	0	−1.92572	0.054139	0.999817	0.99963	1.000003
RELB	0	−1.82117	0.068581	0.999859	0.999708	1.000011
CD2	0	−1.79848	0.072101	0.999872	0.999732	1.000012
TRBV18	−0.007	−1.79285	0.072996	0.992864	0.985121	1.000668
PIM2	0	−1.79045	0.073381	0.999913	0.999819	1.000008
KLHDC7B	0	−1.75477	0.079299	0.999942	0.999877	1.000007
AL606834.2	−0.007	−1.69148	0.090745	0.993121	0.985209	1.001096
TESPA1	−0.001	−1.68354	0.092271	0.999415	0.998734	1.000096
IFNG	−0.003	−1.65036	0.09887	0.996621	0.992622	1.000635
HLA.DQB2	0	−1.6428	0.100424	0.999883	0.999743	1.000023
CCL22	0	−1.62196	0.104813	0.999762	0.999475	1.00005
CD37	0	−1.598	0.110044	0.999891	0.999757	1.000025
IGHG4	0	−1.58194	0.113664	0.999988	0.999974	1.000003
TRBV12.4	−0.007	−1.51643	0.12941	0.993283	0.984668	1.001973
FASLG	−0.001	−1.50584	0.132109	0.998747	0.997118	1.000378
GZMA	0	−1.47864	0.139236	0.999745	0.999406	1.000083
AC004585.1	−0.002	−1.47704	0.139664	0.998327	0.99611	1.000548
TRAV8.4	−0.006	−1.47261	0.140856	0.993756	0.985506	1.002075
ITGAL	0	−1.40573	0.159805	0.999934	0.999842	1.000026
TRAV12.2	−0.004	−1.37904	0.167883	0.99609	0.990559	1.001652
CAMK4	0	−1.32771	0.184273	0.999609	0.999032	1.000186
NAPSB	0	−1.31747	0.187682	0.999835	0.999589	1.000081
CD1A	0	−1.29969	0.193708	0.999669	0.999169	1.000168
AC007569.1	−0.012	−1.29509	0.195289	0.987607	0.969143	1.006423
IGHGP	0	−1.1592	0.246375	0.99997	0.99992	1.000021
RGS1	0	−1.14014	0.254229	0.999972	0.999925	1.00002
SELL	0	−1.08242	0.279068	0.999948	0.999855	1.000042
LINC00494	0.001	1.061845	0.288306	1.000693	0.999414	1.001974
FCRLA	0	−1.01453	0.310331	0.999625	0.9989	1.00035
TRAV1.2	−0.003	−0.66639	0.50516	0.99687	0.98772	1.006104
GPR18	0	−0.57179	0.567466	0.999553	0.99802	1.001087
NME8	0.001	0.569963	0.568703	1.000928	0.99774	1.004127
TRBV13	−0.002	−0.47433	0.635265	0.997881	0.989171	1.006667
IGHG2	0	−0.39298	0.694333	0.999999	0.999994	1.000004
RAB37	0	−0.23861	0.811409	0.999931	0.999364	1.000498

### The IFNG-co-expressed prognostic signature as an independent risk factor of BRCA and definition of a nomogram

**FIGURE 4:**
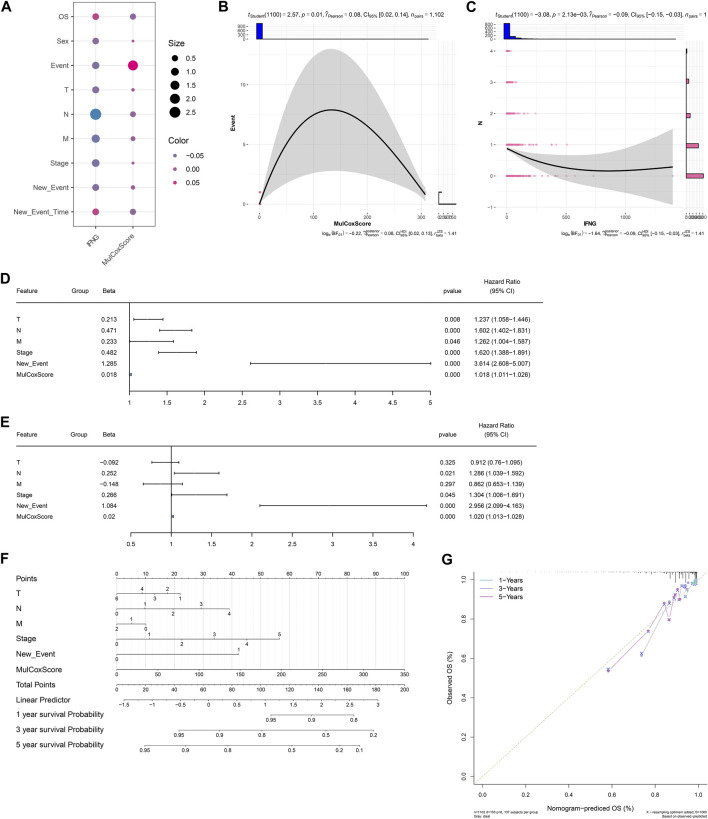
Associations of clinical traits with the IFNG-co-expressed prognostic signature and construction of a nomogram. **(A)** Correlation analysis on IFNG and the IFNG-co-expressed prognostic model with clinical parameters. **(B)** Relationship of the prognostic model *versus* event. **(C)** Relationship of IFNG *versus* N stage. **(D, E)** Uni- or multivariate-cox regression results on the prognostic signature and clinical variables with BRCA survival. **(F)** The nomogram for survival prediction. **(G)** Calibration curves depicting the model-predictive and observed OS.

Next, it was observed that there was a positive connection of the IFNG-co-expressed prognostic signature with event (Figures 4A, B). In addition, IFNG was negatively linked with N stage (Figure 4C). Through considering uni- and multivariate-cox regression results, the prognostic model acted as an independent risk factor of BRCA (Figures 4D, E). The nomogram composed of the prognostic model and clinical traits was defined, and the excellent predictive efficacy was proven by calibration curves (Figures 4F, G).

### Associations of IFNG, and co-expressed AC006369.1, and CCR7 with the TME components

**FIGURE 5:**
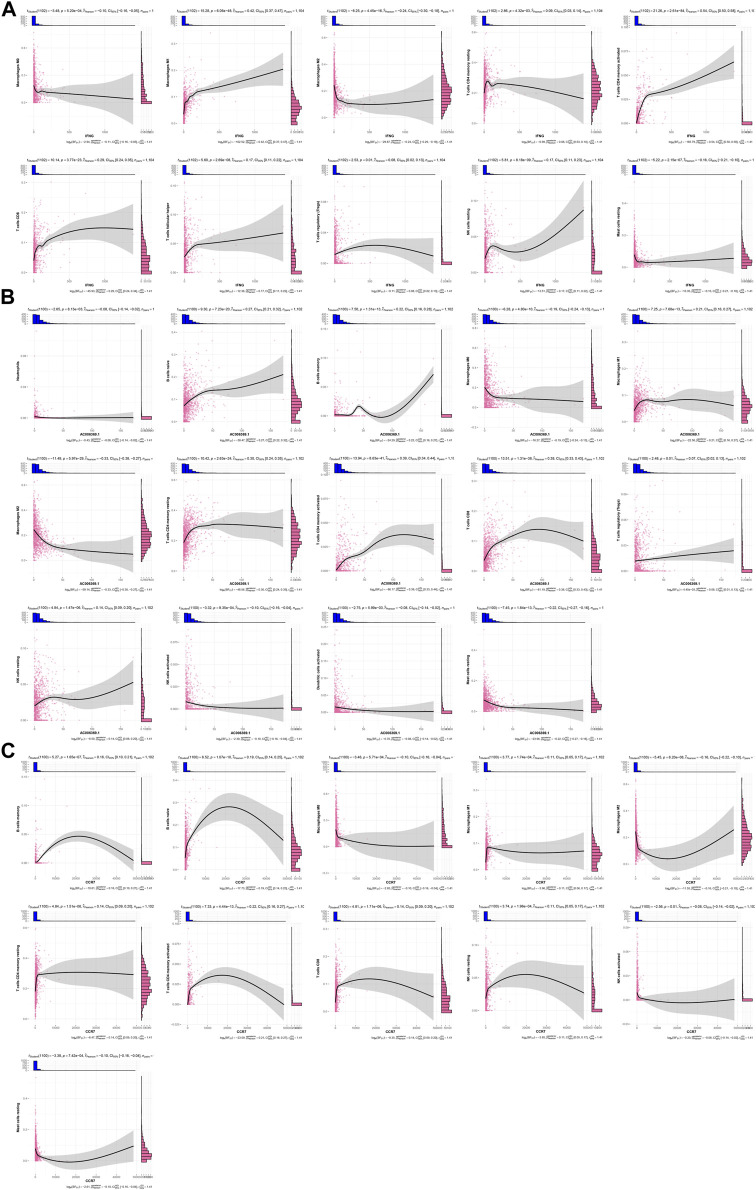
Associations of IFNG, and co-expressed AC006369.1, and CCR7 with the TME components. **(A–C)** Correlation analysis on **(A)** IFNG, **(B)** AC006369.1, and **(C)** CCR7 with the abundance of immune cells within the TME.

IFNG was negatively connected with macrophages M0 and M2, mast cells resting, but was positively linked with macrophages M1, T cells CD4 memory resting and activated, T cells CD8, T cells follicular helper, T cells regulatory (Tregs), and NK cells resting (Figure 5A). This was indicative of the role of IFNG in regulating anti-tumor immunity. In Figure 5B, AC006369.1 presented the negative interactions with neutrophils, macrophages M0 and M2, NK cells activated, dendritic cells activated, and mast cells resting, with positive interactions with B cells naïve and memory, macrophages M1, T cells CD4 memory resting and activated, T cells CD8, Tregs, and NK cells resting. In addition, CCR7 exhibited the positive relationships with B cells naïve and memory, macrophages M1, T cells CD4 memory resting and activated, T cells CD8, and NK cells resting, with negative relationships with macrophages M0 and M2, NK cells activated, and mast cells resting (Figure 5C).

### Interactions of IFNG, and co-expressed genes with immune checkpoints

**FIGURE 6:**
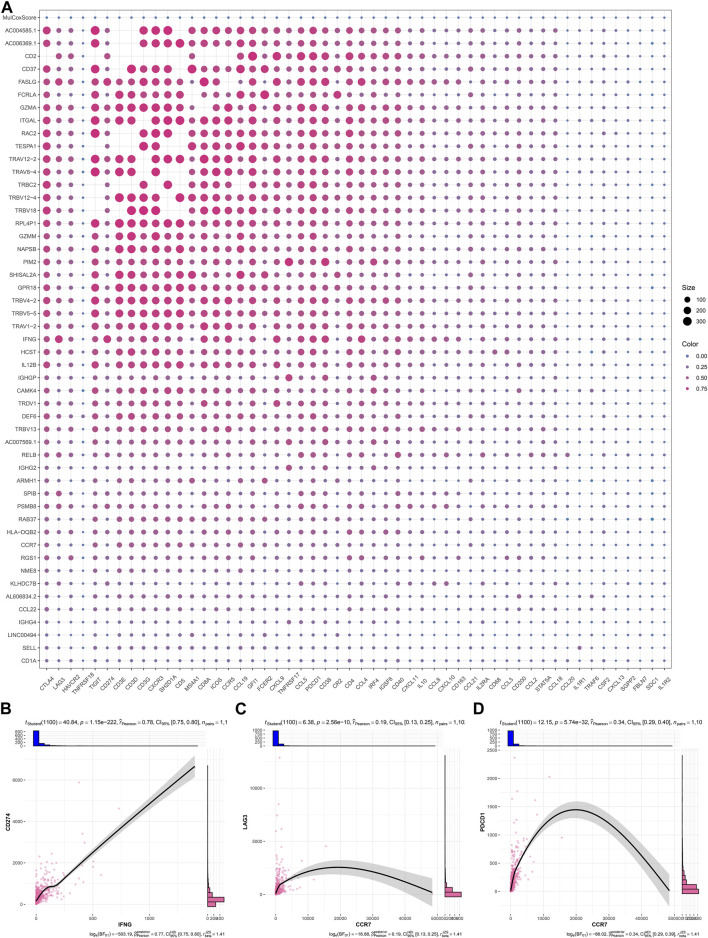
Interactions of IFNG, and co-expressed genes with immune checkpoints. **(A)** Correlation analysis on IFNG, co-expressed genes, and the prognostic signature with immune checkpoints. **(B–D)** Visualization of the relationships of IFNG with CD274, LAG3, and PDCD1.

As illustrated in Figure 6A; Table 2, IFNG, co-expressed genes (notably AC006369.1, and CCR7), and the prognostic model exhibited the positive connections with most immune checkpoint molecules. It was also noted the positive interactions of IFNG with CD274 (PD-1), LAG3, and PDCD1 (Figures 6B–D).

**TABLE 2:** Correlation analyses of IFNG, AC006369.1, and CCR7 with immune checkpoints in BRCA.

Immune checkpoint	IFNG		AC006369.1		CCR7	
	r	p	r	p	r	p
CCL18	0.325517	1.29E-28	0.251434	2.38E-17	0.142041	2.20E-06
CCL19	0.248134	6.33E-17	0.776397	8.92E-223	0.472312	2.59E-62
CCL2	0.286609	2.79E-22	0.234465	3.15E-15	0.132134	1.08E-05
CCL20	0.095234	0.001551	0.004758	0.874644	0.00426	0.887675
CCL21	0.051963	0.084676	0.341864	1.44E-31	0.277394	6.43E-21
CCL3	0.362793	1.29E-35	0.268843	1.06E-19	0.112889	0.000173
CCL4	0.734428	2.24E-187	0.50303	9.82E-72	0.216751	3.49E-13
CCL5	0.678464	1.69E-149	0.652101	1.93E-134	0.316081	5.44E-27
CCL8	0.505601	1.44E-72	0.148589	7.25E-07	0.07855	0.00909
CCR5	0.721719	5.85E-178	0.623922	6.29E-120	0.311697	2.96E-26
CD163	0.450361	3.81E-56	0.227338	2.20E-14	0.102062	0.000691
CD200	0.222103	8.79E-14	0.35874	8.27E-35	0.189568	2.25E-10
CD274	0.776278	1.15E-222	0.345991	2.42E-32	0.168349	1.88E-08
CD38	0.732854	3.52E-186	0.5259	2.12E-79	0.288228	1.59E-22
CD3D	0.577263	6.38E-99	0.873946	0	0.468492	3.30E-61
CD3E	0.543867	7.60E-86	0.887998	0	0.493715	8.93E-69
CD3G	0.67702	1.24E-148	0.773085	1.04E-219	0.42113	1.33E-48
CD4	0.53137	2.54E-81	0.591527	5.57E-105	0.299854	2.49E-24
CD40	0.573782	1.73E-97	0.509643	6.85E-74	0.254698	8.89E-18
CD5	0.507463	3.56E-73	0.843876	1.03E-299	0.480718	8.50E-65
CD68	0.192994	1.05E-10	0.194672	7.15E-11	0.060922	0.043178
CD8A	0.732739	4.30E-186	0.735715	2.33E-188	0.354788	4.94E-34
CR2	0.150807	4.92E-07	0.482392	2.67E-65	0.294252	1.89E-23
CSF2	0.208605	2.67E-12	0.121952	4.93E-05	0.070092	0.019964
CTLA4	0.608277	1.71E-112	0.730355	2.68E-184	0.408044	1.85E-45
CXCL10	0.487788	6.12E-67	0.196638	4.56E-11	0.09579	0.001455
CXCL11	0.527657	5.17E-80	0.295427	1.24E-23	0.147004	9.53E-07
CXCL13	0.072472	0.016117	0.083451	0.005572	0.039526	0.189804
CXCL9	0.739629	2.18E-191	0.62981	7.77E-123	0.327891	4.94E-29
CXCR3	0.614729	1.65E-115	0.808784	7.48E-256	0.424934	1.52E-49
FBLN7	−0.07183	0.017081	0.031189	0.300927	0.018202	0.546117
FCER2	0.114882	0.000132	0.562931	3.97E-93	0.37077	3.06E-37
GFI1	0.627363	1.28E-121	0.719688	1.67E-176	0.365492	3.67E-36
HAVCR2	0.465557	2.29E-60	0.371185	2.51E-37	0.148284	7.64E-07
ICOS	0.631108	1.74E-123	0.67846	1.70E-149	0.406015	5.52E-45
IGSF6	0.554317	8.87E-90	0.46468	4.06E-60	0.197137	4.06E-11
IL10	0.459602	1.09E-58	0.350066	4.05E-33	0.175888	4.15E-09
IL1R1	0.049448	0.100874	0.097552	0.001185	0.039495	0.190162
IL1R2	0.056551	0.060567	0.023964	0.426762	0.010433	0.729378
IL2RA	0.421939	8.40E-49	0.330214	1.91E-29	0.182877	9.62E-10
IRF4	0.420006	2.50E-48	0.498811	2.21E-70	0.283082	9.40E-22
LAG3	0.769047	4.90E-216	0.377368	1.28E-38	0.188985	2.56E-10
MS4A1	0.219143	1.89E-13	0.667616	4.08E-143	0.423381	3.70E-49
PDCD1	0.72552	1.02E-180	0.667504	4.74E-143	0.343997	5.74E-32
SDC1	−0.04353	0.148748	−0.07891	0.008775	−0.05836	0.052782
SGPP2	0.160109	9.12E-08	0.035293	0.241745	0.016745	0.578712
SH2D1A	0.613515	6.18E-115	0.810235	1.76E-257	0.445128	9.67E-55
STAT5A	0.208098	3.02E-12	0.274147	1.89E-20	0.203145	9.96E-12
TIGIT	0.608306	1.66E-112	0.826834	3.57E-277	0.464217	5.50E-60
TNFRSF17	0.366472	2.32E-36	0.493874	7.97E-69	0.24518	1.50E-16
TNFRSF18	−0.02112	0.483628	−0.01387	0.64559	−0.01949	0.518006
TRAF6	0.081821	0.006575	0.076896	0.010663	0.025213	0.403071

### Genetic alterations and DNA methylation of IFNG, and co-expressed genes

**FIGURE 7:**
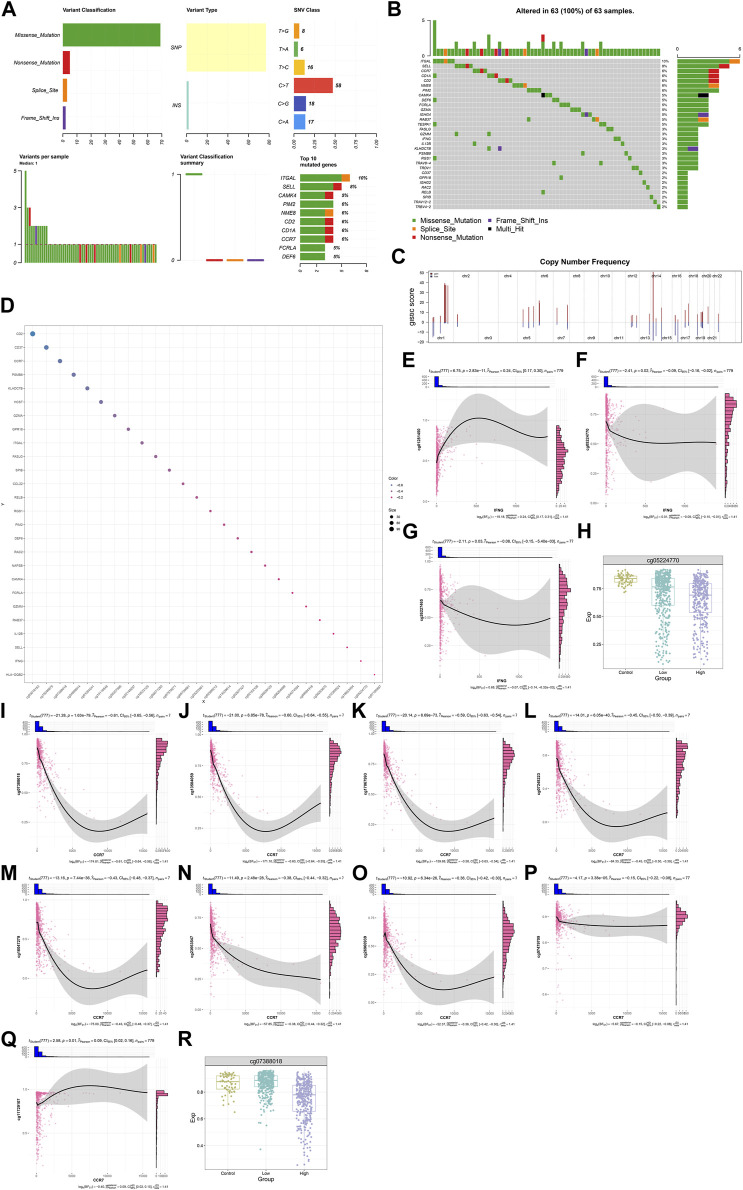
Genetic alterations and DNA methylation of IFNG, and co-expressed genes. **(A)** The summary of somatic mutations in BRCA. **(B)** The mutated frequency of IFNG, and co-expressed genes across BRCA. **(C)** Copy-number frequency of above genes. **(D)** Methylation sites of above genes. **(E–G)** Relationships of IFNG with **(E)** cg01281450, **(F)** cg05224770, and **(G)** cg26227465. **(H)** The beta level of cg05224770 in controls, BRCA with lowly or highly expressed IFNG. (I–Q) Associations of CCR7 with **(I)** cg07388018, **(J)** cg13504059, **(K)** cg17067993, **(L)** cg07248223, **(M)** cg16047279, **(N)** cg23663547, **(O)** cg26960939, **(P)** cg07479709, and **(Q)** cg11729107. **(R)** The beta level of cg07388018 across normal specimens, BRCA with down- or upregulated IFNG.

Most IFNG, and co-expressed genes occurred frequent mutation across BRCA samples, such as CCR7 (6%), and IFNG (3%) (Figures 7A, B). In addition, frequent amplifications were found, which might contribute to their overexpression (Figure 7C). DNA methylation sites were also analyzed (Figure 7D). IFNG expression was positively connected with the beta value of cg01281450, with negative connections with the beta values of cg05224770, and cg26227465 (Figures 7E–G). Among the three CpGs, cg01281450 exhibited the lower beta value in BRCA *versus* controls, with lower value in high *versus* low IFNG expression tumors (Figure 7H). This indicated the contribution of cg05224770 hypomethylation to IFNG upregulation. Moreover, CCR7 expression exhibited the negative interactions with the beta values of cg07388018, cg13504059, cg17067993, cg07248223, cg16047279, cg23663547, cg26960939, and cg07479709, with positive interactions with the beta value of cg11729107 (Figures 7I–Q). Among the CpGs, cg07388018 owned the lower beta value in tumors with IFNG upregulation *versus* controls or tumors with IFNG downregulation (Figure 7R). Thus, hypomethylated cg07388018 possibly resulted in CCR7 overexpression.

### Transcription factors and RNA binding proteins that potentially modulate IFNG and co-expressed genes

**FIGURE 8:**
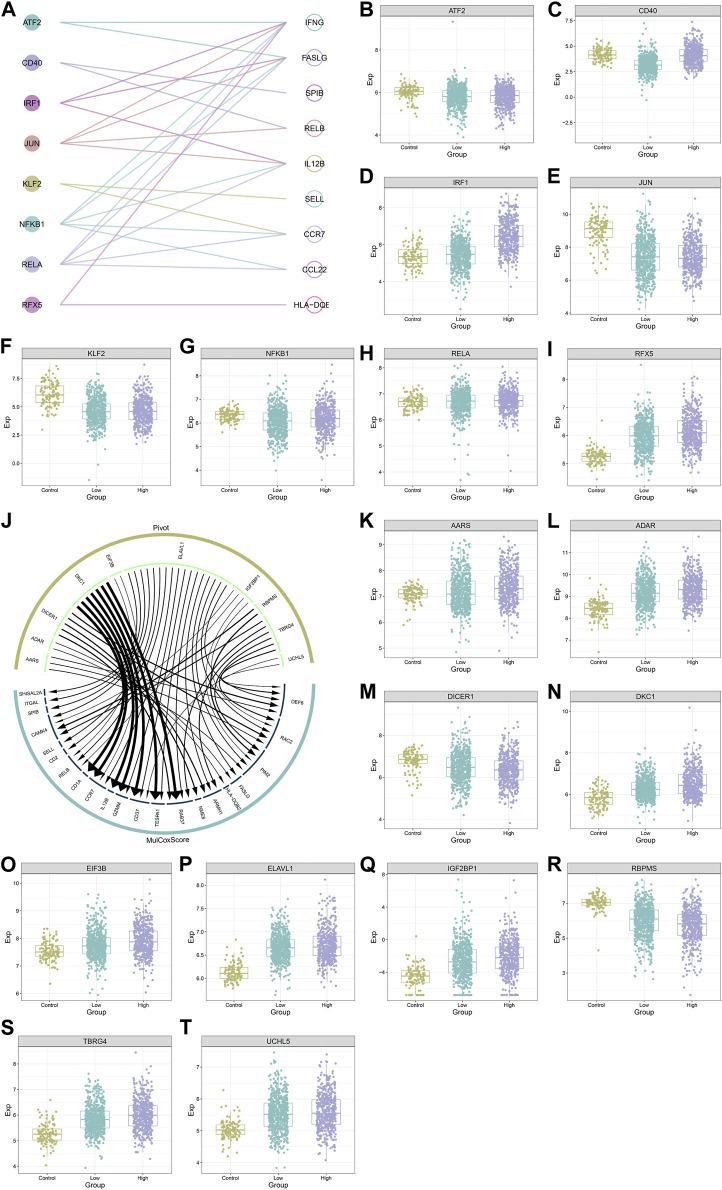
Transcription factors and RNA binding proteins that potentially modulate IFNG and co-expressed genes. **(A)** Interactions of IFNG and co-expressed genes with transcription factors. **(B–I)** The transcript level of **(B)** ATF2, **(C)** CD40, **(D)** IRF1, **(E)** JUN, **(F)** KLF2, **(G)** NFKB1, **(H)** RELA, and **(I)** RFX5 across controls, BRCA with down- or upregulated IFNG. **(J)** The interaction network of IFNG-co-expressed genes with RNA binding proteins. **(K–T)** The transcript level of **(K)** AARS, **(L)** ADAR, **(M)** DICER1, **(N)** DKC1, **(O)** EIF3B, **(P)** ELAVL1, **(Q)** IGF2BP1, **(R)** RBPMS, **(S)** TBRG4, and **(T)** UCHL5 in normal tissues, BRCA with down- or upregulated IFNG.

Figure 8A illustrates eight transcription factors potentially modulating the transcription of IFNG and co-expressed genes, as follows: ATF2 (IFNG, FASLG), CD40 (SPIB, RELB), IRF1 (IL12B, FASLG, IFNG), JUN (FASLG, IL12B, IFNG, RELB), KLF2 (SELL, CCR7), NFKB1 (CCR7, IFNG, IL12B, CCL22, FASLG), RELA (FASLG, IL12B, CCL22, CCR7, IFNG), RFX5 (HLA-DQB2, IFNG). Additionally, these transcription factors exhibited the aberrant expression in BRCA *versus* controls (Figures 8B–I). The heterogeneity in their expression was also found between down- or upregulated IFNG tumors. Ten RNA-binding proteins post-transcriptionally modulated IFNG-co-expressed genes, following AARS (DEF6, RAC2, PIM2, FASLG, HLA-DQB2), DICER1 (ARMH1, RAC2, DEF6, NME8, RAB37), DKC1 (TESPA1, RAB37, CD37, GZMM, IL12B, CCR7, CD1A), EIF3B (CCR7, RELB), ELAVL1 (CD2, RAC2, SELL, ARMH1, CAMK4, DEF6, SPIB, FASLG, CD37, CCR7, TESPA1, RELB, ITGAL, GZMM, RAB37, PIM2, SHISAL2A), IGF2BP1 (PIM2, RELB, CAMK4, RAC2), RBPMS (PIM2, CAMK4), TBRG4 (DEF6, PIM2, ARMH1, CAMK4, RAB37, RAC2), and UCHL5 (DEF6, CD37) (Figure 8J). Except for DICER1, other RNA-binding proteins were upregulated in BRCA (Figures 8K–T).

### MiRNAs and lncRNAs that possibly regulate IFNG and co-expressed genes

**FIGURE 9:**
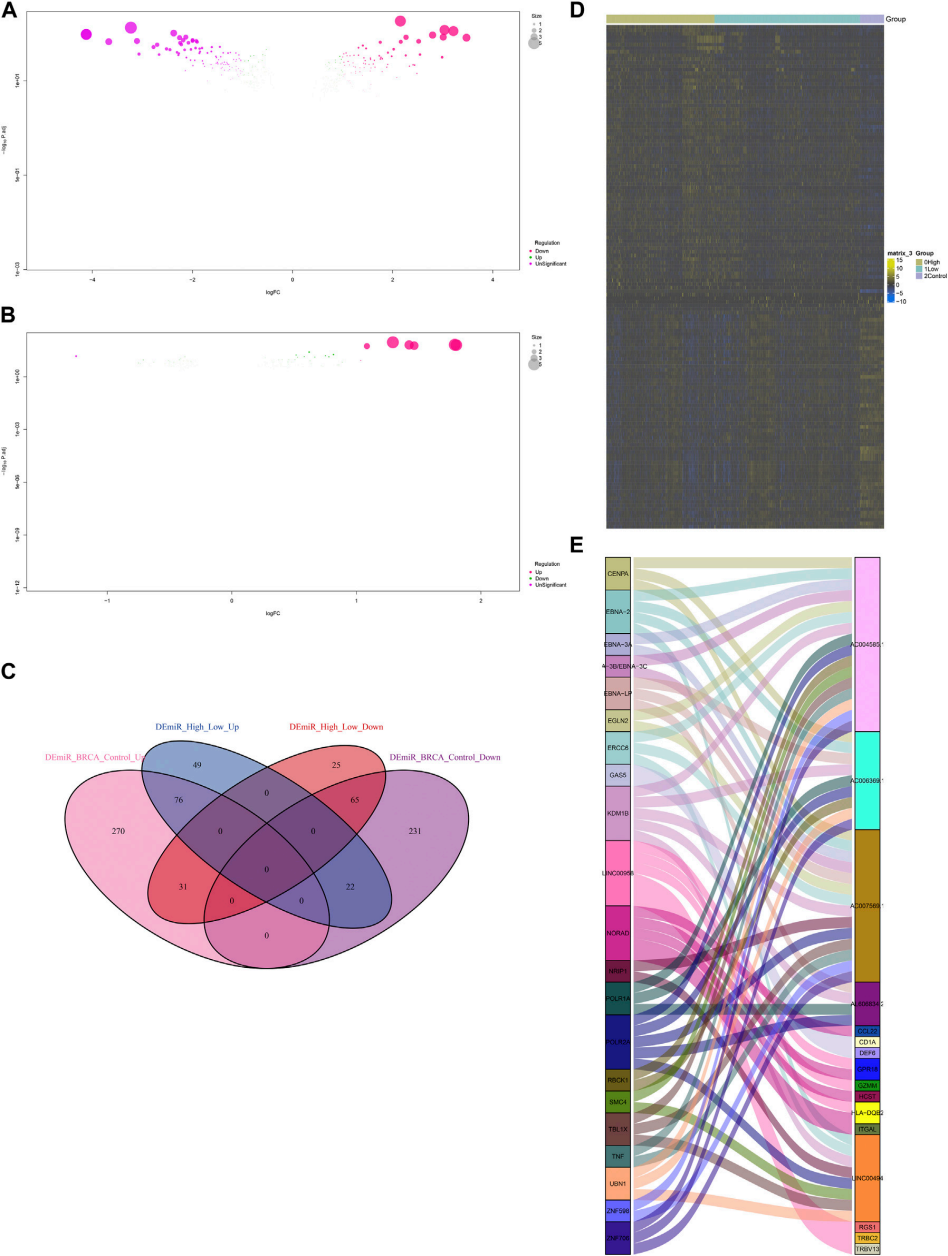
MiRNAs and lncRNAs that possibly regulate IFNG and co-expressed genes. **(A)** MiRNAs with aberrant expression in BRCA *versus* normal specimens. **(B)** MiRNAs with different expression between down- and upregulated IFNG BRCA. **(C)** Venn diagram illustrating the BRCA- and IFNG-relevant miRNAs. **(D)** The expression of above miRNAs across normal tissues, BRCA with down- or upregulated IFNG. **(E)** Correlations of IFNG-co-expressed genes with lncRNAs.

Non-coding RNA-mediated post-transcriptional mechanisms of IFNG and co-expressed genes were also probed. In Figure 9A, 695 miRNAs with aberrant expression were determined in BRCA relative to controls. Additionally, 268 miRNAs exhibited the different expression between lowly and highly expressed IFNG tumors (Figure 9B). Following the intersection, 141 BRCA- and IFNG-relevant miRNAs were selected, which were possibly associated with IFNG expression (Figures 9C, D; Supplementary Table S3). Several lncRNAs were then observed to be potentially interacted with IFNG-co-expressed genes (Figure 9E).

## Discussion

IFNG presented the upregulation in BRCA, as priorly reported (Yaghoobi et al., 2018). Also, the upregulation was associated with favorable OS and RFS outcomes. Thus, IFNG might own the potential as a prognostic marker of BRCA. Two IFNG-co-expressed RNAs (AC006369.1, and CCR7) constituted a Cox regression model for BRCA prognostication. AC006369.1, and CCR7 were aberrantly expressed in BRCA, and in relation to survival outcomes. Similarly, Gu et al. identified AC006369.1 as an IFNG-relevant lncRNA that was connected with prognostic outcomes and the TME in uterine corpus endometrial carcinoma (Gu et al., 2022). Many studies have proven the essential function of CCR7 in BRCA. For instance, CXCL12 facilitates CCR7 ligand-driven BRCA cell invasion and migration towards lymphatic vessels (Hayasaka et al., 2022). Deng et al. reported that site-specific polyplex on downregulated CCR7 increases T cells for hindering lymphatic metastasis of BRCA (Deng et al., 2022). In addition, CCR7 chemokine receptor stimulation can induce rapid but transient dendritic cell migration towards draining lymph nodes, which is crucial for initiating protective immunity and maintaining immune homeostasis (Liu et al., 2019).

IFNG presented the negative connections with macrophages M0 and M2, mast cells resting, with the positive correlations to macrophages M1, T cells CD4 memory resting and activated, T cells CD8, T cells follicular helper, Tregs, and NK cells resting. The interactions of IFNG with such immune cells have been unveiled. For instance, tumor-associated macrophages accelerate metastases as well as hinder T cells. Non-etheless, macrophage polarization is capable of killing malignant cells. IFN-γ can reprogram CD206+ tumor-associated macrophages to inducible iNOS + macrophages in BRCA (Sun et al., 2021). Tregs maintain BRCA progression through manipulating IFN-γ-driven functional reprogramming of myeloid cells (Clark et al., 2020). IFN-γ impairs the cytotoxicity of NK cells via upregulation of PD-L1 on malignant cells as well as PD-1 on NK cells in trastuzumab-resistant HER2-positive BRCA (Zheng et al., 2021). IFN-γ-triggered intermediate monocytes hinder cancer metastasis through activating NK cells (Wang R. et al., 2022). The interactions of IFNG-co-expressed genes (especially AC006369.1 and CCR7) with the TME components were also investigated across BRCA.

Immunotherapy exhibits effective therapeutic potential for long-term cancer regression, but exerts a low response rate owing to insufficient immunogenicity of malignant cells (Tian et al., 2023). IFN-γ is an essential driver of PD1/PD-L1 expression in tumor and host cells. In addition, IFN-γ is capable of upregulating expression of other critical immune suppressive molecules within the TME. Mark Ayers et al. proposed an IFNG-relevant mRNA signature that can predict clinical response to anti-PD-1 therapy (Ayers et al., 2017). Nevertheless, the pleiotropic effects of IFN-γ on immunotherapy have been found, such as immunotherapeutic resistance. IFN-γ-driven adaptive resistance remains one barrier to the improvement in immunotherapy. In the Cucolo et al.‘s study, IFN-γ-driven RIPK1 enhances malignant cell intrinsic as well as extrinsic resistance to immunotherapy (Cucolo et al., 2022). UBR5 facilitates tumor immune escape via elevating IFN-γ-driven PDL1 transcription in BRCA (Wu B. et al., 2022). This work also exhibited the close connections of IFNG with immune checkpoints in BRCA, proving the potential in improving immunotherapy.

The regulatory mechanisms of IFNG and co-expressed genes were further probed. It was found that somatic mutation frequencies of CCR7, and IFNG were separately 6%, and 3%. Frequent amplification also potentially led to their upregulation. Hypomethylated cg05224770 and cg07388018 might associate with IFNG and CCR7 upregulation. IFNG expression can be transcriptionally modulated by ATF2, IRF1, JUN, NFKB1, RELA, and RFX5. Among them, IRF1 has been proven as an IFNG-inducible gene (Qian et al., 2018). IFN-γ-induced IRF-1 attenuates BRCA cell specific growth (Armstrong et al., 2015). RNA-binding proteins (AARS, ADAR, DICER1, DKC1, EIF3B, ELAVL1, IGF2BP1, RBPMS, TBRG4, and UCHL5) and non-coding RNAs also post-transcriptionally affected IFNG and co-expressed genes. The interactions of IFN-γ with ADAR and DICER1 have been partly proven. ADAR (an interferon-inducible RNA-editing enzyme) mitigates IFN signaling in gastric carcinoma through down-regulating STAT1 and IRF9 by miR-302a (Jiang et al., 2020). DICER1 hinders the interferon response in murine embryonic stem cells (Gurung et al., 2021). Invasive micropapillary carcinoma is a rare histological subtype of BRCA with an aggressive phenotype and an undesirable prognosis (Verras et al., 2022a). Invasive micropapillary carcinoma has a high rate of lymphovascular invasion and lymph node metastasis, and has been reported in multiple organs (Verras et al., 2022b; Shi et al., 2022). However, so far, no studies have reported the role of inflammatory cell death-related IFNG and co-expressed RNAs (AC006369.1, and CCR7) in this subtype.

The limitations of our work require to be acknowledged. Despite the close connections of IFNG and co-expressed genes with the TME and immune checkpoint molecules, their roles in anti-tumor immunity need experimental verification. Moreover, further analyses are required for proving the regulatory mechanisms of IFNG and co-expressed genes in BRCA.

## Conclusion

Altogether, this work characterized IFNG and its co-expressed RNAs (notably AC006369.1, and CCR7) as prognostic markers for BRCA individuals, and unveiled their potential as therapeutic targets for the improvement of immunotherapy. Despite this, in-depth experiments will be implemented for proving our conclusions in future research.

## Data Availability

The original contributions presented in the study are included in the article/Supplementary Material, further inquiries can be directed to the corresponding author.

## Abbreviations

BRCA, breast carcinoma; ER, estrogen receptor; HER2, human epidermal growth factor receptor 2; IFN-γ, interferon-γ; NK, natural killer; TME, tumor microenvironment; TCGA, The Cancer Genome Atlas; miRNA, microRNA; WGCNA, weighted correlation network analysis; GO, Gene Ontology; KEGG, Kyoto Encyclopedia of Genes and Genomes; lncRNAs, long non-coding RNAs; OS, overall survival; RFS, recurrence-free survival; Tregs, T cells regulatory.
